# Complete Genome Sequence of *Kluyvera intestini* sp. nov., Isolated from the Stomach of a Patient with Gastric Cancer

**DOI:** 10.1128/genomeA.01184-17

**Published:** 2017-10-26

**Authors:** George Tetz, Maria Vecherkovskaya, Paul Zappile, Igor Dolgalev, Aristotelis Tsirigos, Adriana Heguy, Victor Tetz

**Affiliations:** a Human Microbiology Institute, New York, New York, USA; b Applied Bioinformatics Laboratories, New York University Medical Center, New York, New York, USA; c Department of Pathology, New York University School of Medicine, New York, New York, USA; d Laura and Isaac Perlmutter Cancer Center, New York University School of Medicine, New York, New York, USA; e Genome Technology Center, Division of Advanced Research Technologies, University of New York School of Medicine, New York, New York, USA

## Abstract

We report here an update to the draft genome sequence of *Kluyvera intestini* sp. nov. strain GT-16, generated using MinION long-read sequencing technology. The complete genome sequence of the human-derived strain GT-16 measured 5,768,848 bp. An improved high-quality complete genome sequence provides insights into the mobility potential of resistance genes in this species.

## GENOME ANNOUNCEMENT

*Kluyvera intestini* sp. nov. strain GT-16 is a newly described species that was isolated, using an original genomic workflow, from the stomach of a patient with gastric cancer (1–3). This strain is characterized by numerous virulence factors and antibiotic resistance genes (ARGs) that correspond to those in other clinically significant *Kluyvera* sp. pathogens (4–6). To update the draft genome sequence, we used a combination of Illumina short-read and Oxford Nanopore Technologies (ONT) MinION long-read sequencing technologies (7).

Whole-genome shotgun libraries for Illumina sequencing were prepared as follows: 500 ng of genomic DNA were sheared to 500-bp fragments using a Covaris E220 ultrasonicator, and a library was prepared using the Kapa low-throughput “with bead” preparation kit (Kapa Biosystems, catalog no. KK8231) without PCR amplification. The DNA was sequenced as 300-bp paired-end reads on the Illumina MiSeq platform. DNA was also sequenced using a flow cell system on the ONT MinION sequencer.

Base-calling of the initial ONT MinION FAST5 files was performed with the Metrichor service. The base-called FAST5 files were converted to FASTQ format using poretools (8). Assembly was performed using Canu (9), and nanopolish (10) was used to compute an improved consensus sequence.

*De novo* assembly, combined with manual integration of Nanopore data via LASTZ alignments, yielded two scaffolds of 5,520,555 bp and 248,293 bp, with an average coverage of 150× and a total of 6,098 coding sequences. The DNA-DNA hybridization value showed 99.5% similarity with *K. intestini* sp. nov. GT-16 (GenBank accession no. MKZW00000000), meaning that these genomes belong to the same species (11).

Numerous virulence-related genes, such as those coding hemolysin D, peptidases, permeases, chemotaxis regulators, and exonucleases, were found in *K. intestini* sp. nov. GT-16. High-confidence ARGs (containing over 75% amino acid identity) were identified using the NCBI and Comprehensive Antibiotic Resistance databases (12, 13).

Using complete genome sequencing, we evaluated the mobility potential of the identified ARGs. We identified all mobile genetic elements (MGEs), such as insertion and/or transposable elements, or phages, using the ISFinder, PHAST, and GypsyGenes algorithms (14, 15). The genome was shown to harbor 24 insertion sequence (IS) elements, 2 transposases, 8 resolvases, 11 integrases, and 5 Tn*3* transposons. Next, we merged the outputs to identify all composite elements that were suggested as ARGs and colocalized within the MGEs. The composite elements were determined to be an ARG flanked by two ISs belonging to the same family within a 10-kb region, a transposon with an ARG within a 5-kb region, or a transposable phage.

Overall, the genome of *K. intestini* strain GT-16 harbored 18 ARGs associated with MGEs, including those encoding resistance to tellurium, quaternary ammonium compounds, arsenic, beta-lactams, copper, chloramphenicol, polymyxin, and multidrug resistance (MDR) transporters. This indicates that these ARGs can be involved in horizontal gene transfer (16). Follow-up studies of *K. intestini* and its harbored bacteriophages would enable us to understand its possible pathogenicity and role in cancer (17).

### Accession number(s).

This complete genome sequencing project has been deposited in GenBank under the accession no. MKZW00000000. The version described in this paper is the second version, MKZW02000000.

## References

[1] Tetz G, Tetz V 2016 Draft genome sequence of *Kluyvera intestini* strain GT-16 isolated from the stomach of a patient with gastric cancer. Genome Announc 4(6):e01432-16. doi:10.1128/genomeA.01432-16. 28007864 PMC5180392

[2] Tetz G, Tetz V 2017 Introducing the sporobiota and sporobiome. Gut Pathog 9:38. doi:10.1186/s13099-017-0187-8. 28680484 PMC5493122

[3] Tetz G, Tetz V, Vecherkovskaya M 2016 Genomic characterization and assessment of the virulence and antibiotic resistance of the novel species *Paenibacillus* sp. strain VT-400, a potentially pathogenic bacterium in the oral cavity of patients with hematological malignancies. Gut Pathog 8:6. doi:10.1186/s13099-016-0089-1. 26900405 PMC4761199

[4] Carter JE, Evans TN 2005 Clinically significant *Kluyvera infections*: a report of seven cases. Am J Clin Pathol 123:334–338. doi:10.1309/61XP-4KTL-JYWM-5H35. 15716228

[5] Saul SR, Chen R, Jiang P, Schade M, Jaker M 2014 *Kluyvera cryocrescens* presenting as a complicated urinary tract infection: case report and literature review. Infect Dis Clin Pract 22:e50–e51. doi:10.1097/IPC.0b013e3182a01f35.

[6] Sarria JC, Vidal AM, Kimbrough RC 2001 Infections caused by *Kluyvera* species in humans. Clin Infect Dis 33:E69–E74. doi:10.1086/322686. 11528588

[7] Mikheyev AS, Tin MM 2014 A first look at the Oxford Nanopore MinION sequencer. Mol Ecol Resour 14:1097–1102. doi:10.1111/1755-0998.12324. 25187008

[8] Loman NJ, Quinlan AR 2014 Poretools: a toolkit for analyzing nanopore sequence data. Bioinformatics 30:3399–3401. doi:10.1093/bioinformatics/btu555. 25143291 PMC4296151

[9] Koren S, Walenz BP, Berlin K, Miller JR, Bergman NH, Phillippy AM 2017 Canu: scalable and accurate long-read assembly via adaptive k-mer weighting and repeat separation. Genome Res 27:722–736. doi:10.1101/gr.215087.116. 28298431 PMC5411767

[10] Loman NJ, Quick J, Simpson JT 2015 A complete bacterial genome assembled *de novo* using only nanopore sequencing data. Nat Methods 12:733–735. doi:10.1038/nmeth.3444. 26076426

[11] Auch AF, von Jan M, Klenk HP, Göker M 2010 Digital DNA-DNA hybridization for microbial species delineation by means of genome-to-genome sequence comparison. Stand Genomic Sci 2:117–134. doi:10.4056/sigs.531120. 21304684 PMC3035253

[12] Jia B, Raphenya AR, Alcock B, Waglechner N, Guo P, Tsang KK, Lago BA, Dave BM, Pereira S, Sharma AN, Doshi S, Courtot M, Lo R, Williams LE, Frye JG, Elsayegh T, Sardar D, Westman EL, Pawlowski AC, Johnson TA, Brinkman FS, Wright GD, McArthur AG 2017 CARD 2017: expansion and model-centric curation of the comprehensive antibiotic resistance database. Nucleic Acids Res 45:D566–D573. doi:10.1093/nar/gkw1004. 27789705 PMC5210516

[13] Tatusova T, DiCuccio M, Badretdin A, Chetvernin V, Ciufo S, Li W 2013 Prokaryotic genome annotation pipeline. *In* Beck J, Benson D, Coleman J, Hoeppner M, Johnson M, Maglott D, Mizrachi I, Morris R, Ostell J, Pruitt K, Rubinstein W, Sayers E, Sirotkin K, Tatusova T (ed), The NCBI handbook, 2nd ed. NCBI, Bethesda, MD.

[14] Siguier P, Perochon J, Lestrade L, Mahillon J, Chandler M 2006 ISfinder: the reference centre for bacterial insertion sequences. Nucleic Acids Res 34:D32–D36. doi:10.1093/nar/gkj014. 16381877 PMC1347377

[15] Zhou Y, Liang Y, Lynch KH, Dennis JJ, Wishart DS 2011 PHAST: a fast phage search tool. Nucleic Acids Res 39:W347–W352. doi:10.1093/nar/gkr485. 21672955 PMC3125810

[16] Stokes HW, Gillings MR 2011 Gene flow, mobile genetic elements and the recruitment of antibiotic resistance genes into Gram-negative pathogens. FEMS Microbiol Rev 35:790–819. doi:10.1111/j.1574-6976.2011.00273.x. 21517914

[17] Tetz GV, Ruggles KV, Zhou H, Heguy A, Tsirigos A, Tetz V 2017 Bacteriophages as potential new mammalian pathogens. Sci Rep 7:7043. doi:10.1038/s41598-017-07278-6. 28765534 PMC5539208

